# Locoregional recurrence patterns in women with breast cancer who have not undergone post-mastectomy radiotherapy

**DOI:** 10.1186/s13014-020-01637-w

**Published:** 2020-09-04

**Authors:** Xuran Zhao, Yu Tang, Shulian Wang, Yong Yang, Hui Fang, Jianyang Wang, Hao Jing, Jianghu Zhang, Guangyi Sun, Siye Chen, Jing Jin, Yongwen Song, Yueping Liu, Bo Chen, Shunan Qi, Ning Li, Yuan Tang, Ningning Lu, Hua Ren, Yexiong Li

**Affiliations:** Department of Radiation Oncology, National Cancer Center/National Clinical Research Center for Cancer/Cancer Hospital, Chinese Academy of Medical Sciences and Peking Union Medical College, 17 Panjiayuannanli, Chaoyang District, Beijing, 100021 China

**Keywords:** Breast neoplasm, Mastectomy, Locoregional recurrence, Risk factors

## Abstract

**Background:**

To analyze the patterns of locoregional recurrence in breast cancer patients after mastectomy.

**Methods:**

The retrospective study included 7073 women with breast cancer without post-mastectomy radiotherapy: 4604 (65.1%) had pT1–2 N0 disease (low risk); 2042 (28.9%), pT1–2 N1 (intermediate risk); and 427 (6.0%), pT3–4 and/or pN2–3, or pT1–2 N1 after neoadjuvant chemotherapy (high risk). The distribution of cumulative locoregional recurrence was analyzed. The local recurrence and regional recurrence rates were estimated by the Kaplan-Meier method, and differences were compared with the log-rank test. Multivariate analysis was performed using Cox logistic regression analysis.

**Results:**

In the median follow-up of 63.0 months, 469 patients had locoregional recurrence: chest wall recurrence in 238 (50.7%) cases, supraclavicular/infraclavicular nodes in 236 (50.3%) cases, axilla in 92 (19.6%), and internal mammary nodes in 50 (10.7%) cases. The 5-year local recurrence and regional recurrence rates were 2.5 and 4.4%, respectively. Subgroup analysis of the three risk groups and five molecular subtypes (luminal A, luminal B-Her2 negative, luminal B-Her2 positive, Her2-enriched, and triple negative) also showed that the chest wall and supraclavicular/infraclavicular nodes were the most common recurrence sites. Age, tumor location, T stage, N stage, and hormone receptor status were independent prognostic factors for both local recurrence and regional recurrence (*p* < 0.05).

**Conclusions:**

The chest wall and supraclavicular/infraclavicular nodes are common sites of locoregional recurrence in breast cancer, irrespective of disease stage or molecular subtype, and the prognostic factors for local recurrence and regional recurrence are similar. Therefore, chest wall and supraclavicular/infraclavicular nodes irradiation should always be considered in post-mastectomy radiotherapy.

## Background

Postmastectomy radiotherapy (PMRT) is an important strategy for the locoregional management of breast cancer, as it can reduce the risk of locoregional recurrence (LRR) and decrease breast cancer mortality [1]. Previous randomized studies have shown that chest wall and comprehensive regional nodal irradiation result in a decrease in LRR and an improvement in overall survival in patients with node-positive or T3–4 disease [2–4]. However, the recurrence patterns have changed with the advances in diagnostic technologies and therapeutic approaches, and as a result, the optimal radiation target volume is now under debate. Some studies have shown that the risk of regional nodal recurrence in patients with 1–3 positive nodes is relatively low [5–9], but it is unclear whether comprehensive locoregional radiotherapy is essential for improving survival, or whether delivering radiation to a more limited target volume may achieve a comparable outcome. In addition, several studies have examined whether high-risk patients with T1–2N0 disease are suitable for PMRT, but the optimal radiation target volume is unclear [10–14].

Understanding the local and regional recurrence patterns in more recent cohorts of patients could help in making decisions about radiotherapy, such as the ideal target volume. Although some studies have investigated the patterns of LRR after mastectomy, the results were limited either by their small sample size, or by the type of population and time period studied. Further, there is little information about LRR patterns after modern systemic therapy, such as standard hormone therapy and anthracycline- or taxane-based chemotherapy, although there is evidence that the modern therapies provide superior outcome to traditional regimens for node-positive patients [15–17]. Additionally, different molecular subtypes are associated with different prognoses, so treatment regimens are typically personalized to the needs of individual patients [18]. However, the association of molecular subtypes with LRR is unclear. In this study, we seek to fill in all these information gaps by analyzing the patterns of LRR in a large series of patients with breast cancer who underwent mastectomy along with modern treatment regimens, and by investigating whether locoregional failure was related to disease stage or molecular subtypes.

## Methods

**Fig. 1:**
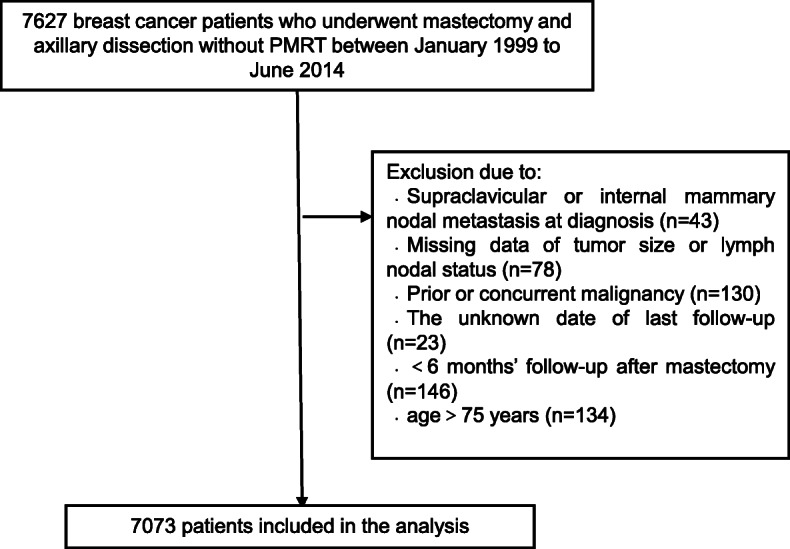
Flow diagram showing patient selection

This study was approved by our Institutional Review Board (approval number 15–057/984). Patients with pathologically confirmed breast cancer who underwent mastectomy between January 1999 and June 2014 and met the following criteria were eligible: 18–75 years of age, no PMRT, no evidence of distant dissemination or supraclavicular internal mammary nodal metastasis at diagnosis, no prior or concurrent malignancy, and ≥ 6 months of follow-up after mastectomy (Fig. 1).

The molecular subtypes were constructed according to the ER, PR, and HER2 status, and the tumors were graded into five categories: (1) luminal A: ER+ or PR+, HER2-, and grade 1 or 2; (2) Luminal B-Her2 negative: ER+ or PR+, HER2-, and grade 3; (3) Luminal B-Her2 positive: ER+ or PR+, and HER2+; (4) Her2-enriched: ER-, PR- and HER2+; and (5) triple-negative breast cancer (TNBC): ER-, PR-, and HER2-. Tumor grade was used to construct the molecular subtypes, because the Ki-67 index was only available for 1436 (20.3%) patients.

Follow-up data were obtained from hospital records or from correspondence directly with the patient or their family. All recurrences were confirmed by pathologic or radiographic evidence. LRR included local recurrence (LR) and/or regional recurrence (RR). LR was defined as recurrence in the ipsilateral chest wall, and RR was defined as recurrence involving the ipsilateral axillary, supraclavicular/infraclavicular, or internal mammary lymph nodes that was detected during follow-up, regardless of whether distant metastasis occurred earlier, later, or simultaneously. Recurrence at any other site was considered as distant metastasis. The cumulative LR, RR, and LRR rates were estimated by the Kaplan-Meier method, and the differences were compared with the log-rank test. The recurrence rates were calculated from the date of surgery. Multivariate analysis was performed using Cox logistic regression analysis. Significant variables (as indicated by a significance value of *P* < 0.05) identified from the univariate analysis were included in the Cox regression model. All *P* values were two-tailed, and a value of less than 0.05 was considered to be indicate significance. All statistical analyses were carried out using the SPSS Package for Windows, version 24.0 (SPSS Inc., Chicago, IL, USA).

## Results

The present study cohort comprised 7073 patients. The patient, tumor, and treatment characteristics are summarized in Table 1. The median age was 50 years (range, 19–75 years). Axillary lymph node dissection was performed in 6734 (95.2%) patients, and 339 (4.8%) patients with pN0 disease underwent sentinel node biopsy alone. The median number of axillary lymph nodes dissected was 19 (range, 1–63). According to the seventh edition of the American Joint Committee on Cancer (AJCC) Staging System for breast cancer, patients were divided into three groups based on the tumor burden: the low-risk group comprising 4604 (65.1%) patients who had pT1–2 N0 disease, the intermediate-risk group comprising 2042 (28.9%) patients who had pT1–2 N1 disease, and the high-risk group comprising 427 (6.0%) patients (370 with pT3–4 or pN2–3 disease, and 57 with pT1–2 N1 disease after neoadjuvant chemotherapy). In 5330 cases, there was sufficient information for determination of the molecular subtype: 2397 (45.0%), luminal A; 573 (10.8%), luminal B-Her2 negative; 810 (15.2%), luminal B-Her2 positive; 583 (10.9%), Her2 enriched; and 967 (18.1%), triple negative (TN).

**Table 1:** Local and regional recurrence rates stratified by patient, tumor, and treatment characteristics

Characteristic	No. of Patients (%)	5-year LR (%)	HR (95%CI)	P	5-year RR (%)	HR (95%CI)	P
Age				0.005			<0.001
>45 years	4864 (68.8)	2.2	1.000		3.5	1.000	
≤ 45 years	2209 (31.2)	3.1	1.450 (1.118–1.879)		6.4	1.863 (1.492–2.325)	
Location				<0.001			<0.001
Other quadrants	5211 (73.7)	1.9	1.000		3.6	1.000	
Inner quadrant	1686 (23.8)	3.6	1.729 (1.303–2.293)		6.0	1.663 (1.305–2.120)	
Unknown	176 (2.5)						
Pathologic type				0.210			0.058
Others	893 (12.6)	1.7	1.000		2.9	1.000	
Ductal	6150 (87.0)	2.6	1.309 (0.858–1.996)		4.6	1.445 (0.986–2.118)	
Unknown	30 (0.4)						
T stage^a^				<0.001			<0.001
T1–2	6967 (98.5)	2.2	1.000		4.0	1.000	
T3–4	106 (1.5)	24.2	13.018 (8.813–19.231)		31.0	8.961 (6.075–13.216)	
N stage^a^				<0.001			<0.001
N0	4604 (65.1)	1.6	1.000		2.5	1.000	
N1	2142 (30.3)	2.9	1.940 (1.459–2.580)		5.9	2.922 (2.271–3.761)	
N2	173 (2.4)	9.1	5.549 (3.381–9.108)		12.2	6.124 (3.825–9.804)	
N3	154 (2.2)	17.6	14.513 (9.488–22.199)		33.1	18.467 (12.827–26.587)	
No. of node dissected				0.035			0.007
≥ 10	6485 (91.7)	2.4	1.000		4.3	1.000	
<10	588 (8.3)	4.9	1.598 (1.029–2.481)		7.2	1.652 (1.145–2.384)	
Tumor grade				0.001			0.001
1	409 (5.8)	0	1.000		1.7	1.000	
2	3678 (52.0)	2.1	4.748 (1.171–19.244)		3.7	2.403 (1.062–5.440)	
3	1713 (24.2)	3.2	7.004 (1.715–28.607)		5.6	3.503 (1.534–8.001)	
Unknown	1273 (18.0)						
Lymphovascular invasion				0.252			0.062
No	6668 (94.3)	2.4	1.000		4.2	1.000	
Yes	354 (5.0)	4.7	1.357 (0.803–2.291)		6.9	1.507 (0.977–2.325)	
Unknown	51 (0.7)						
Hormone receptor status				<0.001			<0.001
Positive	5212 (73.7)	1.9	1.000		3.3	1.000	
Negative	1745 (24.7)	4.4	1.906 (1.467–2.476)		7.8	1.990 (1.585–2.498)	
Unknown	116 (1.6)						
HER2 status				0.196			0.001
Negative	4536(64.1)	2.2	1.000		3.8	1.000	
Positive	1400 (19.8)	3.1	1.241 (0.894–1.722)		6.5	1.564 (1.204–2.031)	
Unknown	1137 (16.1)						
Ki67				0.553			0.082
≥ 14%	724 (10.2)	4.8	1.000		7.5	1.000	
<14%	712 (10.1)	6.1	1.143 (0.734–1.780)		10.2	1.378 (0.958–1.981)	
Unknown	5637 (79.7)						
Molecular subtypes							
Luminal A	2397 (33.9)	1.3	1.000	<0.001	2.5	1.000	<0.001
Luminal B-Her2 negative	573 (8.1)	2.6	2.132 (1.303–3.486)		5.3	1.920 (1.264–2.917)	
Luminal B-Her2 positive	810 (11.5)	3.0	1.689 (1.032–2.765)		5.8	1.944 (1.329–2.842)	
Her2-enriched	583 (8.2)	3.4	2.029 (1.231–3.343)		7.5	2.322 (1.564–3.447)	
Triple-negative	967 (13.7)	4.9	2.711 (1.842–3.991)		7.3	2.279 (1.626–3.194)	
Unknown	1743 (24.6)						
Endocrine therapy^b^				0.075			0.782
Yes	4400 (84.4)	1.4	1.000		2.9	1.000	
No	569 (10.9)	2.7	1.535 (0.954–2.470)		2.7	0.931 (0.563–1.541)	
Unknown	243 (4.7)						
Chemotherapy				<0.001			<0.001
No	1662 (23.5)	1.3	1.000		1.9	1.000	
Yes	5373 (76.0)	2.8	2.424 (1.561–3.765)		5.2	2.979 (1.993–4.451)	
Unknown	38 (0.5)						
Anti-Her2 target therapy^c^				0.079			0.415
Yes	321 (22.9)	1.0	1.000		6.1	1.000	
No	1067 (76.2)	3.6	2.437 (0.871–6.814)		6.7	1.271 (0.713–2.264)	
Unknown	12 (0.9)						

Abbreviations: *HER2* human epidermal growth factor receptor 2

^a^ For patients who did not receive neoadjuvant chemotherapy, we used pathological stage because it is more accurate than clinical stage. For patients who received neoadjuvant chemotherapy, we used whichever stage was higher (clinical or pathological) to reflect the actual tumor burden

^b^ Only hormone-receptor positive patients included

^c^ Only Her2 positive patients included

Out of the 7073 patients in the cohort, 5373 (76.0%) patients received neoadjuvant and/or adjuvant chemotherapy with a median of 6 cycles (range, 1–12). Among them, 5219 (97.1%) received adjuvant chemotherapy and 154 (2.9%) received neoadjuvant chemotherapy. The chemotherapy regimens included anthracycline and/or taxane in 4669 (86.9%) patients, while other regimens were used in the remaining 704 (13.1%) patients. Among the 5212 patients with hormone receptor-positive disease, 4400 (84.4%) received adjuvant endocrine therapy. The median duration of endocrine therapy was 47 months (range, 1–160 months). In 1400 patients with Her2-positive disease, 321 (22.9%) received trastuzumab therapy.

**Fig. 2:**
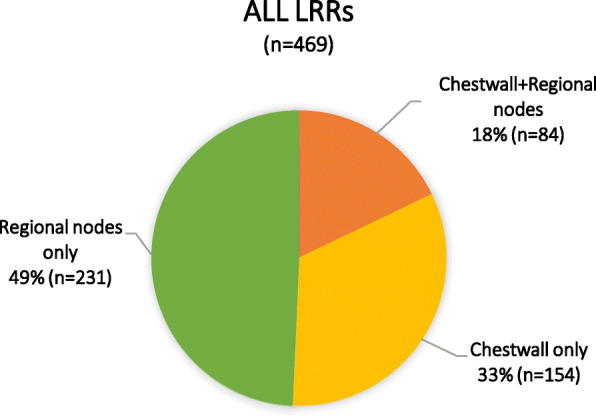
Overall distribution of cumulative locoregional recurrences

**Fig. 3:**
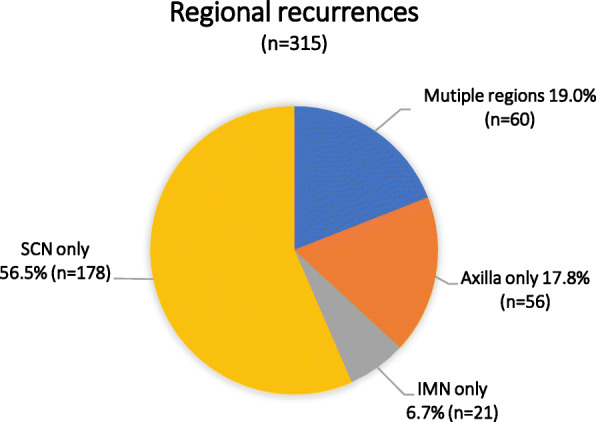
Distribution of regional recurrences

The median follow-up was 63.0 months (range, 6.0–194.6 months), and 469 (6.6%) patients developed LRR. Among them, 154 (32.8%) had LR alone, 231 (49.3%) had RR alone, and 84 (17.9%) had both LR and RR (Fig. 2). Figure 3 showed the regional failure patterns for patients with RR. The 5-year cumulative incidence of LRR was 6.4%. The distribution of LRR sites in the 469 patients was as follows: chest wall in 238 (50.7%) cases, of which 170 (71.4%) cases were pathologically confirmed; supraclavicular/infraclavicular nodes (SCN) in 236 (50.3%) cases, of which 131 (55.5%) cases were pathologically confirmed; axilla in 92 (19.6%) cases, of which 45 (48.9%) cases were pathologically confirmed; and internal mammary nodes (IMN) in 50 (10.7%) cases, of which 20 (40.0%) cases were pathologically confirmed. The distribution of LRR according to risk group and molecular subtype is shown in Table 2 and Table 3. Table 4 shows the distribution of LRR between Her2-positive patients who did and did not receive targeted therapy (trastuzumab therapy), and between hormone receptor-positive patients who did and did not receive endocrine therapy.

**Table 2:** The incidence and sites of LRR by risk group

Risk group	Total No.	LRR	CW	SCN	Axilla	IMN
		No. (%)	No. (%)	No. (%)	No. (%)	No. (%)
Low Risk	4606	170 (3.7)	92 (54.1)	68 (40.0)	27 (15.9)	27 (15.9)
Intermediate Risk	2042	185 (9.1)	83 (44.9)	110 (59.5)	37 (20.0)	20 (10.8)
High Risk	427	114 (26.7)	63 (55.3)	58 (50.9)	28 (24.6)	3 (2.6)
Total cohort	7073	469 (6.6)	238 (50.7)	236 (50.3)	92 (19.6)	50 (10.7)

Abbreviations: *CW* chest wall; *SCN* supraclavicular/infraclavicular nodes; *IMN* internal mammary nodes; *LRR* locoregional recurrence

**Table 3:** The distribution of LRR by intrinsic molecular subtype

Molecular Subtypes	Total No.	LRR	CW	SCN	Axilla	IMN
		No. (%)	No. (%)	No. (%)	No. (%)	No. (%)
Luminal A	2397	105 (4.4)	47 (44.8)	50 (47.6)	20 (19.0)	9 (8.6)
Luminal B-Her2 negative	573	42 (7.3)	24 (57.1)	23 (54.8)	11 (26.2)	7 (16.7)
Luminal B-Her2 positive	810	58 (7.2)	25 (43.1)	30 (51.7)	13 (22.4)	6 (10.3)
Her2-enriched	583	50 (8.6)	23 (46.0)	32 (64.0)	8 (16.0)	7 (14.0)
Triple-negative	967	100 (10.3)	58 (58.0)	50 (50.0)	21 (21.0)	12 (12.0)
Total cohort	5330	355 (6.7)	177 (49.9)	185 (52.1)	73 (20.6)	41 (11.5)

**Table 4:** The distribution of LRR by targeted and endocrine therapy in Her2-positive and hormone receptor-positive patients

Characteristic	Total No.	LRR	CW	SCN	Axilla	IMN
		No. (%)	No. (%)	No. (%)	No. (%)	No. (%)
Her2 positive	1400	109 (7.8)	49 (45.0)	63 (57.8)	21 (19.3)	14 (12.8)
Targeted therapy	321	16 (5.0)	4 (25.0)	9 (56.3)	2 (12.5)	4 (25.0)
No targeted therapy	1067	92 (8.6)	44 (47.8)	53 (57.6)	19 (20.7)	10 (10.9)
Hormone receptor positive	5212	281 (5.4)	139 (49.5)	134 (47.7)	57 (20.3)	25 (8.9)
Endocrine therapy	4400	210 (4.8)	95 (45.2)	101 (48.1)	40 (19.0)	23 (11.0)
No endocrine therapy	569	30 (5.3)	21 (70.0)	13 (43.3)	9 (30.0)	2 (6.7)

**Fig. 4:**
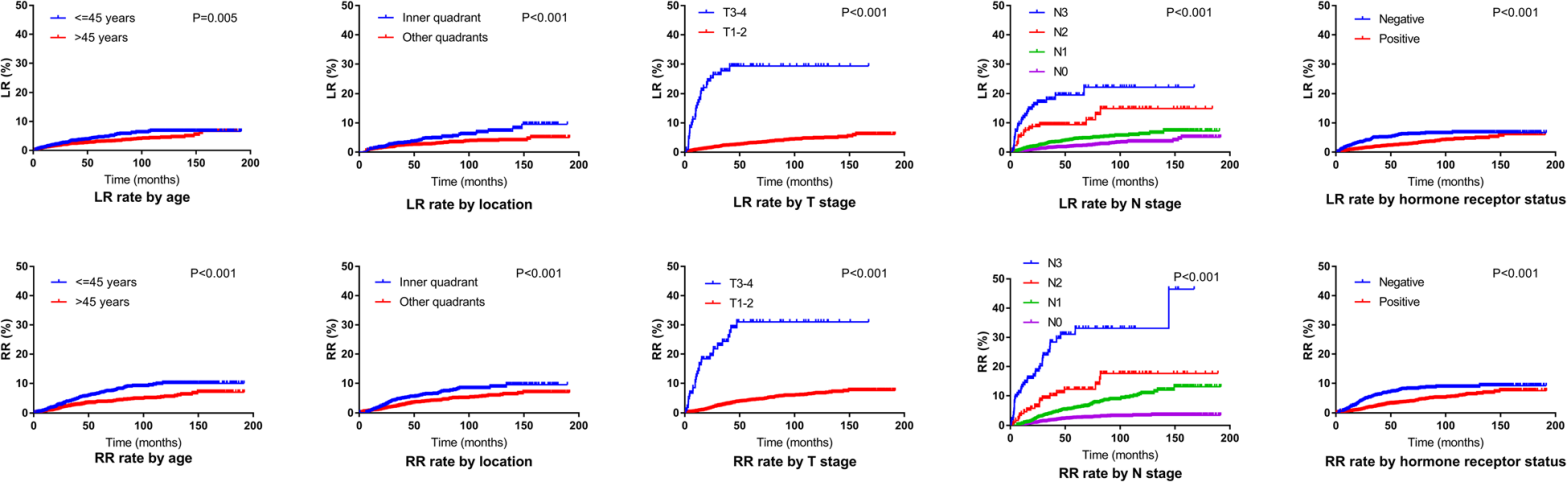
Local and regional recurrence curves according to different prognostic factors for the entire cohort

A total of 238 patients (3.4%) developed LR, and the 5-year cumulative LR rate was 2.5%. The median interval from surgery to LR was 52.4 months (range, 5.9–191.9 months). Further, 315 patients (4.5%) developed RR, and the 5-year cumulative RR rate was 4.4%. The median interval from surgery to RR was 29.5 months (range, 0.6–149.4 months). The results of univariate analysis of the association of clinical variables with the risk of LR and RR are shown in Table 1. The significant variables identified in the univariate analysis were used for multivariate analysis, which showed that age ≤ 45 years, location of the tumor in the inner quadrant, T3–4 stage disease, N1–3 stage disease, and hormone receptor negative were associated with increased risk of both LR and RR (Table 5). Since the SCN was the most common site of regional recurrence, we also investigated the prognostic factors of recurrence in the SCN, to assess whether statistical significance was still present for the variables analyzed. The univariate and multivariate analyses showed that the independent prognostic factors of recurrence in the SCN were the same as those of RR (supplementary Table 1). The LR and RR rates of patients with respect to different prognostic factors are displayed in Fig. 4.

**Table 5:** Multivariate analysis of prognostic factors for local recurrence and regional recurrence

Variable	LR		RR	
	HR (95% CI)	P	HR (95% CI)	P
Age, y		0.001		<0.001
>45	1.000		1.000	
≤ 45	1.688 (1.232–2.313)		2.061 (1.560–2.723)	
Tumor location		<0.001		0.001
Other quadrants	1.000		1.000	
Inner quadrant	2.059 (1.488–2.851)		1.684 (1.251–2.268)	
No. of axillary nodes dissected		0.428		0.299
≥ 10	1.000		1.000	
<10	1.337 (0.652–2.741)		1.407 (0.738–2.682)	
T Stage		<0.001		<0.001
T1–2	1.000		1.000	
T3–4	7.381 (4.097–13.296)		3.585 (2.025–6.349)	
N Stage		<0.001		<0.001
N0	1.000		1.000	
N1	2.070 (1.455–2.944)		3.215 (2.321–4.453)	
N2	2.716 (1.241–5.947)		5.169 (2.752–9.709)	
N3	9.871 (5.223–18.656)		12.636 (7.215–22.131)	
Tumor grade		0.144		0.110
1	1.000		1.000	
2	3.719 (0.913–15.147)		1.433 (0.626–3.282)	
3	4.109 (0.992–17.010)		1.881 (0.807–4.384)	
Hormone receptor status		0.002		0.007
Positive	1.000		1.000	
Negative	1.741 (1.236–2.454)		1.536 (1.123–2.102)	
Her2 status				0.535
Negative			1.000	
Positive			1.105 (0.806–1.515)	
Chemotherapy		0.256		0.247
No	1.000		1.000	
Yes	1.374 (0.794–2.380)		1.371 (0.804–2.337)

## Discussion

The present study reports the patterns of LRR in a large cohort of post-mastectomy breast cancer patients who received modern treatment regimens, and the findings show that the SCN and chest wall recurrence rates were similar in the patient cohort, irrespective of the disease stage or molecular subtype, but axillary or IMN recurrence was relatively rare. This finding is slightly different from those of previous studies, as shown in Table 6 [5, 11–14, 19–33], which show that the chest wall is the most common site of LRR. The decrease in chest wall recurrence is probably attributable to advances in surgical techniques that have made it possible to use thinner flaps for surgery. In the present cohort analyzed, 95.2% of the patients had undergone axillary dissection, only 4.8% of the patients had undergone sentinel lymph node biopsy, although 65.1% of the patients had N0 disease. The usage of axillary dissection in the present study was similar to that of previous studies shown in Table 6. In addition, a randomized trial has established that similar low risk of axillary recurrence was found in N0 patients regardless of axillary procedures [34]. Therefore, axillary dissection may not be a factor in reducing the rate of recurrence recorded in axilla, with a consequent higher percentage of relapses recorded in the SCN. Whereas advances in radiology have resulted in an increase in the sensitivity of detection of SCN recurrence [35], and improvements in the effectiveness of systemic therapy have reduced the risk of LRR and might also change the LRR patterns, which might be the explanation for the high percentage of relapses recorded in the SCN.

**Table 6:** Comparison of LRR patterns between previous studies and the present study in patients without PMRT

Study	Treatment decade	No. of patients	Tumor stage	N stage	Axillary dissection	The median number of ALNs dissected	Adjuvant chemotherapy (regimens)	Endocrine therapy	Anti-Her2targeted therapy	LRR type (No. of patients)	CW	SCN	Axillary	IMN	Regional	Local + Regional
Truong, P. T. et al. [12]	1989–1999	1505	60.7%T1 39.3%T2	N0	100%	10	20.8%	48.4%	–	First (89)	68.5%	19.1%	24.7%	3.4%	31.5%	15.7%
Abi-Raad, R. et al. [11]	1980–2004	1136	75.3%T1 24.7%T2	N0	90%	14	14.6%	56.6%	–	First (58)	72.4%	13.8%	10.3%	6.9%	27.6%	1.7%
Hastings, J. et al. [13]	1994–2004	1259	T1	N0	–	11	24.2%	77.7%	–	Accumulated (33)	75.8%	9.1%	27.3%	0.0%	24.2%	12.1%
Li, J. L. et al. [14]	2001–2008	353	45.6%T1 54.4%T2	N0	100%	–	100%	unless contraindicated or intolerant	–	First (39)	53.8%	48.7%	10.3%	2.6%	46.2%	15.4%
Sharma, Ranjna et al. [19]	1997–2002	1019	78.8%T1 21.2%T2	73.9%N0 26.1%N1	68.1%	16	41.6% (94.7% anthracycline- and/or taxane-based)	87.2%	–	Accumulated (23)	52.1%	39.2%	4.3%	17.4%	47.8%	4.3%
Jwa, E. et al. [20]	2002–2011	390	58.5%T1 41.5%T2	78.7%N0 21.3%N1	–	6	54.4% (100% anthracycline- and/or taxane-based)	95.0%	8.9%	Accumulated (12)	50.0%	33.3%	33.3%	8.3%	–	–
Asaga, S. et al. [21]	–	428	49.5%T1 51.5%T2	N1	98.6%	16	67% (100% anthracycline- and/or taxane-based)	–	22.0%	First (20)	50.0%	35.0%	20.0%	5.0%	–	5.0%
wallgren et al. [22]	1978–1993	5352	T1–3N0–3 (41%T1)	24%N0 45%N1 31%N2/3	–	≥8	for high-risk patients (CMF)	Most post-menopausal patients with N+	–	First (1138)	53%	26%	13%	1%	–	7% (multiple sites)
Katz et al. [23]	1975–1994	1031	II-III (80%T1–2 10%T3 10%Tx)	14%N0 45%N1 26%N2 15%N3	100%	17	100% (96% anthracycline- based)	68.2%	–	Accumulated (179)	68%	47%	14%	8%	–	–
Rangan, A. M. et al. [24]	1980–1991	217	88.5%T1–2 7.8%T3 3.7%Tx	76%N1 24%N2	100%	14	100% (82%CMF)	50%	–	First (42)	52.4%	40.5%	14.3%	7.1%	47.6%	9.5%
Taghian, A et al. [25]	1984–1994	5758	–	51.4%N1 32.2%N2 16.1%N3	100%	16	100% (90.3% anthracycline- based)	–	–	Isolated (715)	56.9%	22.6%	11.7%	<1%	–	–
Nielsen et al. [26]	1982–1990	1545	II-III	–	–	≥5	55.4%CMF	–	–	Isolated (456)	57%	14%	45%	0%	43%	11%
Macdonald et al. [27]	1990–2004	165	T1–2N1	–	100%	14	62%	>90%	–	Accumulated (13)	77%	23%	38%	–	–	–
Trovo et al. [28]	1999–2005	150	I-II	54%N0 8%N1mic 37%N1a	97.3%	18	38.7% (75.9% anthracycline- based)	>90%	–	Accumulated (17)	64.7%	64.7%	–	–	–	29.4%
Muhsen, S et al. [29]	1995–2006	924	T1–2N1	–	94%	18	86% (76% anthracycline- and/or taxane-based)	100%	41%	First (56)	62.5%	25.0%	17.9%	5.4%	37.5%	8.9%
Chen et al. [30]	2001–2007	390	T1–2N0–1 Triple-negative	78.7%N0 21.3%N1	100%	–	86.4% (79.2% anthracycline- based)	–	–	Accumulated (31)	41.9%	29.0%	0.0%	6.5%	–	22.6%
Kuo et al. [31]	–	115	91.5%T1–2	–	–	–	–	–	–	Isolated (115)	65%	25%	15%	0%	–	–
Skinner et al. [32]	1994–2006	159	91.5%T1–2 13.2%T3–4 4.4%Tx	50.9%N0 32.7%N1 8.8%N2 5.0%N3	89%	–	61.6%	69.6%	–	Isolated (159)	77%	27%	13%	4%	–	–
Ma et al. [33]	2005–2013	235	91.5%T1–2	54.9%N0 33.6%N1 11.5%N2/3	100%	12	92.8% (83.9% anthracycline- and/or taxane-based)	94.6%	6.7%	Isolated (235)	48.5%	44.7%	14.0%	18.3%	51.5%	13.2%
Strom, E. A. et al. [5]	1975–1994	1031	83%T1–2 10%T3 7%Tx	14%N0 45%N1 41%N2/3	100%	17	doxorubicin-based	–	–	Accumulated (180)	67%	43%	12%	–	53%	–
Current study	1999–2014	7073	65.1%T1–2N0 29.7%T1–2N1 5.2%III	65.1%N0 30.3%N1 4.6%N2/3	95.2%	19	75.6% (86.9% anthracycline- and/or taxane-based)	84.4%	22.9%	Accumulated (469)	50.7%	50.3%	19.6%	10.7%	67.2%	17.9%

The chest wall has been documented as the most common site of LRR so far, and it is generally included in the target volume when PMRT is administered. Regional nodal irradiation, especially SCN irradiation, is widely used for patients with heavy axillary nodal burden, for example, patients with four or more positive nodes. In this study, 26.7% of the patients in the high-risk group developed LRR: chest wall recurrence, 55.3%; SCN recurrence, 50.9%. The high recurrence rate in this group is consistent with the findings of several large-scale studies, which recommend both chest wall and SCN irradiation in these patients [6, 22, 23, 25, 36].

It is unclear whether nodal irradiation can be omitted in those with a lower axillary nodal burden (e.g., those with less than four positive nodes). Among the intermediate-risk patients in this study, 9.1% developed LRR: SCN recurrence in 59.5% of the cases and chest wall recurrence in 44.9% of the cases. However, in previous two small series focusing on T1–2N1 disease, the most common recurrence site was the chest wall (50–77%), and SCN recurrence accounted for 23–35% of all recurrences [21, 27]. Similarly, Karlsson et al. reported that the 10-year recurrence rate for the chest wall, SCN, and axilla was 10.3, 2.6, and 4.8%, respectively, for N1 patients who did not undergo PMRT [37]. In addition, some studies have suggested that regional nodal irradiation should be omitted in N1 patients, because of the low recurrence rate [38] or lack of positive outcome [39]. Further, according to the SUPREMO study [40], which is a randomized Phase III trial assessing the role of chest wall irradiation in women with intermediate-risk breast cancer following mastectomy, regional nodal irradiation was optional. In contrast, in the recent MA20 and EORTC 22922–10925 randomized controlled trials that included 85 and 43.1% patients with N1 disease, respectively, additional SCN and IMN irradiation significantly improved disease-free survival [41, 42]. The findings of these two trials seem to corroborate our findings, which also indicated that SCN should be covered in PMRT for N1 breast cancer. This recommendation is important, as this recurrence pattern is representative of the more recent trends in LRR in post-mastectomy breast cancer patients.

Among the low-risk patients (pT1–2 N0 disease) in this study, 3.7% developed LRR: 54.1% in the chest wall and 40.0% in the SCN. A recent study revealed a similar distribution of LRR: 53.8% chest wall recurrence and 48.7% SCN recurrence [14]. In contrast, some previous studies have shown that chest wall recurrence accounted for 68.5–75.8%, while SCN recurrence only accounted for 9.1–19.1% of all recurrences [11–13]. Further, in the study by Yildirim, all 14 LRRs were located in the chest wall [43]. Thus, recent findings have shown that the SCN might also be an important region to be covered in PMRT, and therefore, the traditional notion that the chest wall is the only site to be irradiated in pT1–2 N0 disease is being challenged.

It has been reported that the Her2-enriched and TN subtypes of breast cancer are the most susceptible to locoregional failure [44, 45]. Further, patients with the TN subtype had a significantly higher risk of RR than those with the other subtypes, while no significant difference was found in the risk of LR [46–48]. Additionally, a German study showed that for all first local, regional, bone, and visceral recurrences of the Luminal A, Luminal B-Her2 negative, Luminal B-Her2 positive, Her2-enriched, and TN subtypes, LR accounted for 13.8, 20.0, 1.1, 6.4, and 36.0% recurrences, respectively, while RR accounted for 21.1, 2.7, 9.5, 12.9, and 6.0% recurrences, respectively [46]. The luminal subtypes were associated with bone recurrence, while the Her2-enriched and TN subtypes were associated with visceral recurrence [49]. Previous investigations generally did not distinguish between patients who underwent breast-conserving surgery and mastectomy, or between patients who did and did not undergo radiotherapy, and none of them specifically focused on LRR patterns based on molecular subtypes. This study tries to answer these questions: we found that the distribution of LRR sites was similar among the five molecular subtypes (the chest wall and SCN were the most common sites). Further, even in Her2-positive patients and hormone receptor-positive patients, the most common LRR sites were the chest wall and SCN, irrespective of whether they underwent anti-HER2-targeted therapy (in the former group) or endocrine therapy (in the latter group). This finding indicates that PMRT must also cover SCN, irrespective of the molecular subtype.

In this study, we found that the prognostic factors for LR and RR were similar; this means that it is necessary to cover both local and regional areas when administering PMRT. Very few studies have examined LR and RR separately. Several studies showed that age [30], T stage [25, 26], pathological type (ductal carcinoma vs. others) [26], and fascia invasion [26] was only associated with LR, and the number of positive nodes (3 vs. 1–2) [30], tumor grade [25], resection of only a few nodes (< 8) [25], and extracapsular invasion [25] was only associated with RR or axillary recurrence. These factors are mostly similar to those identified as being associated with both LR and RR in the present study; therefore, they should be considered when trying to predict locoregional failure in this group of patients.

The limitations of this study should be acknowledged. The main limitation is its retrospective design. Further, only half of the LRRs were confirmed by pathological examination. Despite these limitations, the sample was quite large, and to the best of our knowledge it is the first one to compare the LRR patterns among different disease stages and different molecular subtypes. In this study, we investigated the prognostic factors of LR and RR separately to find if regional nodal irradiation can be individualized, the results demonstrated the necessity to cover both local and regional area when PMRT was indicated.

## Conclusion

In summary, the findings of this study clearly show that the SCN region is as common as the chest wall as a recurrence site, irrespective of tumor stage or molecular subtype. Based on the findings, we recommended that SCN also be covered in all patients who undergo PMRT. Further, the relatively low incidence of axillary and IMN recurrence does not justify the routine application of radiotherapy to these regions.

## Supplementary information

**Additional file 1: Table S1.** The univariate and multivariate analysis of prognostic factors for SCN recurrence

## Data Availability

The datasets used during the current study are available from the corresponding author on reasonable request.

## Abbreviations

LRR: Locoregional recurrence
PMRT: Postmastectomy radiotherapy
LR: Local recurrence
RR: Regional recurrence
SCN: Supraclavicular/infraclavicular nodes
IMN: Internal mammary nodes
TNBC: Triple-negative breast cancer
IHC: Immunohistochemistry
FISH: Fluorescence in situ hybridization
CW: Chest wall
DM: Distant metastasis
