# Supplementary material for: Locoregional recurrence patterns in women with breast cancer who have not undergone post-mastectomy radiotherapy
Source: Radiat Oncol. 2020 Sep 4;15:212. doi: 10.1186/s13014-020-01637-w (PMC7487762; doi:10.1186/s13014-020-01637-w)
Supplement: Supplementary file 1 — Additional file 1: Table S1. The univariate and multivariate analysis of prognostic factors for SCN recurrence [file 13014_2020_1637_MOESM1_ESM.docx]

Supplementary Table 1. The univariate and multivariate analysis of prognostic factors for SCN recurrence

| Variable | Univariate analysis | | Multivariate analysis | |
| --- | --- | --- | --- | --- |
|  | 5-year SCN recurrence rate (%) | P | HR (95% CI) | P |
| Age |  | ＜0.001 |  | ＜0.001 |
| ＞45 years | 2.7 |  | 1.000 |  |
| ≤45 years | 4.8 |  | 1.963 (1.413-2.725) |  |
| Location |  | ＜0.001 |  | 0.002 |
| Other quadrants | 2.7 |  | 1.000 |  |
| Inner quadrant | 4.5 |  | 1.74 (1.228-2.465) |  |
| Unknown |  |  |  |  |
| Pathologic type |  | 0.029 |  | 0.696 |
| Others | 1.6 |  | 1.000 |  |
| Ductal | 3.5 |  | 1.259 (0.398-3.985) |  |
| Unknown |  |  |  |  |
| T stage* |  | ＜0.001 |  | 0.032 |
| T1-2 | 3.1 |  | 1.000 |  |
| T3-4 | 19.0 |  | 2.285 (1.076-4.855) |  |
| N stage* |  | ＜0.001 |  | ＜0.001 |
| N0 | 1.7 |  | 1.000 |  |
| N1 | 4.6 |  | 3.595 (2.452-5.271) |  |
| N2 | 9.4 |  | 4.510 (1.983-10.257) |  |
| N3 | 26.7 |  | 15.48 (8.122-29.502) |  |
| No. of node dissected |  | 0.203 |  |  |
| ≥10 | 3.3 |  |  |  |
| ＜10 | 4.2 |  |  |  |
| Tumor grade |  | 0.007 |  | 0.308 |
| 1 | 1.4 |  | 1.000 |  |
| 2 | 2.8 |  | 1.173 (0.471-2.922) |  |
| 3 | 4.2 |  | 1.513 (0.595-3.852) |  |
| Unknown |  |  |  |  |
| Lymphovascular invasion |  | 0.077 |  |  |
| No | 3.2 |  |  |  |
| Yes | 5.5 |  |  |  |
| Unknown |  |  |  |  |
| Hormone receptor status |  | ＜0.001 |  | 0.003 |
| Positive | 2.4 |  | 1.000 |  |
| Negative | 6.3 |  | 1.720 (1.195-2.474) |  |
| Unknown |  |  |  |  |
| HER2 status |  | 0.001 |  | 0.494 |
| Negative | 2.8 |  | 1.000 |  |
| Positive | 4.9 |  | 1.137 (0.786-1.646) |  |
| Unknown |  |  |  |  |
| Ki67 |  | 0.108 |  |  |
| ≥14% | 5.6 |  |  |  |
| ＜14% | 7.2 |  |  |  |
| Unknown |  |  |  |  |
| Molecular subtypes |  | ＜0.001 |  |  |
| Luminal A | 1.8 |  |  |  |
| Luminal B-Her2 negative | 3.8 |  |  |  |
| Luminal B-Her2 positive | 3.9 |  |  |  |
| Her2-enriched | 6.3 |  |  |  |
| Triple-negative | 5.7 |  |  |  |
| Unknown |  |  |  |  |
| Endocrine therapy† |  | 0.963 |  |  |
| Yes | 2.1 |  |  |  |
| No | 2.1 |  |  |  |
| Unknown |  |  |  |  |
| Chemotherapy |  | ＜0.001 |  | 0.197 |
| No | 1.3 |  | 1.000 |  |
| Yes | 3.9 |  | 1.552 (0.796-3.025) |  |
| Unknown |  |  |  |  |
| Anti-Her2 target therapy‡ |  | 0.204 |  |  |
| Yes | 4.1 |  |  |  |
| No | 5.2 |  |  |  |
| Unknown |  |  |  |  |

Abbreviations: SCN= supraclavicular/infraclavicular nodes; HER2 = human epidermal growth factor receptor 2

* For patients who did not receive neoadjuvant chemotherapy, we used pathological stage because it is more accurate than clinical stage. For patients who received neoadjuvant chemotherapy, we used whichever stage was higher (clinical or pathological) to reflect the actual tumor burden.

† Only hormone-receptor positive patients included.

‡ Only Her2 positive patients included.
